# Defining and Characterizing Temporary Childbirth Migration in India

**DOI:** 10.1007/s11113-025-09947-1

**Published:** 2025-03-21

**Authors:** Nadia G. Diamond-Smith, Rutuja Patil, Dhiraj Agarwal, Rachel Murro, Shrish Raut, Sanjay Juvekar, Alison M. El Ayadi

**Affiliations:** 1 Department of Epidemiology and Biostatistics, University of California San Francisco, San Francisco, CA, USA; 2 Vadu Rural Health Program, KEM Hospital Research Centre, Pune, India; 3 Department of Obstetrics & Gynecology, Bharati Vidyapeeth Deemed to be University Medical College, Pune, India; 4 Department of Obstetrics, Gynecology, & Reproductive Sciences, University of California, San Francisco, USA

**Keywords:** South Asia, Migration, Data quality, Measurement

## Abstract

Women returning to their natal homes for pregnancy, delivery, and postpartum is common and understudied in South Asia, with important implications for maternal and newborn health policies, as well as data quality and interpretation. Using data from 1252 women residing in a Health and Demographic Surveillance Site in Maharashtra, India we explore timing, duration and associated socio-economic factors with Temporary Childbirth Migration (TCM). Our overall goal is to develop a definition of temporary childbirth migration and situate it within demographic migration theory. Most (80%) of women migrated for over 1 month in the last trimester of pregnancy, with a sizeable proportion (22%) departing immediately after delivery. Socio-demographic factors were not associated with migrating during pregnancy; migrating postpartum was associated with younger age and higher education. Based on these findings, we propose a definition of Temporary childbirth Migration as a form of migration from husbands to natal homes and back, for at least one month duration, with departure and return at any time in the perinatal period. Given the potentially large number of women moving location for an extended duration in every pregnancy (in a country of over 1.4 billion), programs providing services to pregnant women and newborns should take this phenomenon into consideration. Additionally, data collection efforts at the clinical and household level should understand that women’s place of delivery or receipt of prenatal or postnatal services may differ from her normal place of residence.

## Introduction

In India, as in much of South Asia, patrilocal norms still drive marriage patterns, and women often move to their husband’s family’s home at the time of marriage (Khalil & Mookerjee, 2018; Palriwala & Uberoi, 2005). While some marriages are within the same village, many women move far from their natal (parent’s) home, to different villages, districts, or even states for marriage (Fulford, 2012). Past studies have looked at the impact of co-residence (nuclear vs joint families) and strength of ties with (and distance to) the natal home on maternal and infant health behaviors and outcomes (Allendorf, 2013; Bloom et al., 2001; Coffey et al., 2022). In the South Asian context women returning to their natal home during pregnancy and staying for the childbirth and into the postpartum period is a common practice, however, this traditional practice is under- described in the demographic, sociological and health literature. Characterizing this phenomenon is important not only to understand this behavior and how it fits into our definition of migration, but also for delineating important health and social consequences, as well as health system solutions.

One recent study using data from two Indian states (Bihar and Madhya Pradesh) found that one-third of women returned to their natal home at some point during pregnancy, for childbirth, or postpartum (Diamond-Smith et al., 2024). Another study of a migrant urban population in Mumbai, India found that about two-thirds of migrant women returned to their natal home for delivery (Gawde et al., 2016). In China, where a practice known as “peiyue care” involves women returning in the second trimester, an estimated 25% of internal migrant workers engage in childbirth return. This has been linked to lower socioeconomic status, decreased assimilation, fewer antenatal care visits, and delayed antenatal care initiation (Ding et al., 2020; Zong et al., 2018). Aside from these few studies, we identified no quantitative studies on temporary childbirth migration, and it has yet to be described in detail in terms of timing, duration, and predictors, or heterogeneity across geography or social groups. Of note, aside from the first study mentioned, the other prior studies all looked at this practice among migrants, not among the general population of women.

Fitting what we know about this phenomenon into the current migration literature and theory is challenging. Existing frameworks on migration describe permanent or temporary residence change for economic, political, environmental or social reasons, with residence change for visits to relatives or medical treatment purposively excluded (International Organization for Migration, 2019). The limitations of current definitions in this area are criticized, with calls for expansion of typologies to a level of nuance and flexibility capable of better capturing the relevant individual and population-level factors affecting migrant experiences. However, other authors conceptualize migration more broadly, referring to any move away from the regular place of residence (Turan et al., 2016). There is a literature on short term, temporary and long-term migration patterns, however different definitions are operationalized. India’s National Sample Survey (NSS) defines short term migrants as those who migrate for between 30 −180 days, with a focus on labor migration (Coffey et al., 2015). Other definitions, including by the European Union and United Nations, and most Health and Demographic Surveillance systems, of short-term migration use the cut off of 3 months, with some going up to 12 months. Another concept, which is perhaps most relevant, is “temporary migration” and is defined as “migration for a specific motivation and purpose with the intention to return to the habitual residence after a limited period of time” (Smith-Greenaway & Madhavan, 2015). With this in mind, for the purposes of this paper we refer to women’s temporary relocation to their natal home during the peripartum period as “temporary childbirth migration.” However, one of the goals of this analysis and paper is to determine the appropriate terminology for this practice and how it should be defined.

Past literature on temporary migration generally, but especially in India, focuses on labor migration. Female migration is common in India, however, it is not commonly for labor. Women make up 83% of India’s permanent internal migrants, and 84% of those are marriage migrants (Rao & Finnoff, 2015). Marriage migration refers to women who marry someone in a different place, and therefore migrate at the time of marriage to permanently move to another place to live with their husband. Women also migrate for other reasons such as economic opportunity; however, women only represent 1.1% of labor migration, consistent with the generally low female labor force participation in India (20.8%) (Afridi et al., 2018; Chatterjee & Desai, 2017; International Labor Organization, n.d.). While there has been research on women’s labor migration and marriage migration, there is acknowledgement of other forms of family migration in India for women, but very little detailed research on other reasons women in India migrate.

One important implication of temporary childbirth migration is the possibility that it could be biasing current population health surveillance approaches. For example, women’s perinatal health classification may be incorrectly attributed to natal homes (women give birth in a location that is not their primary place of residence), leading to inappropriate inference and planning due to lack of accounting for temporary childbirth migration. Population-representative household surveys may also miss women visiting their natal home during pregnancy, potentially underreporting pregnancies and recent births. The Indian National Family Health Survey (NFHS) and the District Level Household Survey in India, the two main sources of demographic data especially related to maternal and child health, do not ask about migration patterns during and after pregnancy (temporary childbirth migration). The structure of the surveys do not allow us to measure exactly where women were at each month leading up to or following birth, and thus, we are unable to precisely know where a woman is seeking care at important times in her own and her infant’s life course. Furthermore, data collected from health facilities may be recording information about women who do not reside locally, thus inferences about the continuum of care or geographically-relevant predictors of health outcomes, may not be valid.

Aside from impacting the quality and biasing our interpretation of demographic and health data, temporary childbirth migration could be impacting health outcomes. One pathway is through disrupting the continuum of care. The basis of information provision, care, and linkages to pregnancy and postpartum health care for rural and semi-rural women in India (which is still the majority of women) is through a network of community health workers (CHWs). In qualitative interviews conducted with Indian CHWs, one CHW said *Many times pregnant women are left [out] because she goes to her parent’s place in the seventh month* and further said that, given a shortage of resources, when women came into the CHW’s village to be in their natal homes in pregnancy, the CHWs could not provide them essential services (Diamond-Smith et al., 2024). Not only do CHWs provide care, but they also collect data that is used to help direct health care resources. Thus, without accounting for temporary childbirth migration, the health system itself may not be providing adequate resources to all women, where they are needed. On the flip side, many women might return to their natal homes for social and emotional support and to be able to rest more, thus it could supersede the health care system and actually lead to better health outcomes for women.

This study aims to determine the magnitude, timing, duration and socio-demographic heterogeneity of Temporary Childbirth Migration in a general, rural population of women in one state of Western India.

## Methods

### Ethical Approval

Ethical approval was obtained from the KEM Hospital Research Centre institutional Ethics committee (letter KEMHRC/RVM/EC-1899 dated 29th September 2022) and the University of California, San Francisco, and all participants provided written informed consent before participating.

### Study Area and Population

This study was conducted within the Vadu Health and Demographic Surveillance System (Vadu HDSS) in Western Maharashtra, India. The Vadu HDSS has been longitudinally monitoring a population of 22 villages since 2002, covering approximately 180,000 individuals. Every year, the Vadu HDSS collects data on pregnancies, births, marriages, migrations, deaths and assesses causes of deaths using verbal autopsies. The average annual number of births in the Vadu HDSS area is 1998. Vadu HDSS is primarily a rural area located approximately 30 kms from Pune city. Data was collected from November, 2022–March, 2023.

### Sample Size and Sample Selection

We recruited 1252 women (from 22 villages across HDSS) who had given birth within the last year of the prior round of data collection (out of the 1775 total births in our study site in the year prior to data collection). The participants were randomly selected from the entire surveillance area, with proportions in each village corresponding to the number of births in each of those villages. Thus, larger villages with more births were overrepresented in our sample. Only women who were usually living in the HDSS were included (not those there visiting their natal home) and more than one woman per household could be included if eligible. Sample size was based on limitations in budget.

### Study Tool Development

Study tools were developed by rigorous literature review and consultations among the investigative team and external experts. The study tool aimed to collect retrospective data on temporary childbirth migration. They were translated in local language (Marathi) and pre-tested before use within the study. Trained Field Research Assistants (FRAs) administered written informed consent before enrolling study participants. Once enrolled, the FRA collected data using structured study questionnaire which included sections to record details of their pregnancy, migration timing, duration, access to care, and reasons for migration or non-migration. Data were collected using android devices with a data collection application designed by the Vadu HDSS IT team, utilizing the open-source platform Survey Solutions. FRAs read the questions to the respondent and inputted the data.

Details of the measures analyzed here are below; additional demographic and socioeconomic status of the women were obtained by linking the temporary childbirth migration survey with routine, ongoing data collected from the HDSS data.

### Measures

#### Temporary Childbirth Migration

Women who had delivered in the last year were asked if they returned to their natal (parents) home for more than 2 weeks at any point in their pregnancy and post-partum period. If they answered yes, they were asked for the date of their departure to their natal home and the date of return. From this we calculated the overall duration of migration, and within certain times in the perinatal period (pregnancy, at the time of delivery, postpartum). Specifically, we made a variable of any migration longer than 1 month, and also made a variable for temporary childbirth migration defined as migrating for 3 months or longer. We also asked women the name and location of their natal village and how long it took them to travel there. From this we can calculate the distance to the natal village from the husband’s village. Distance to natal home was made into a categorical variable of < 5 km (km), 5 to < 20 km, 20 to < 50 km, 50 to < 100 km and over 100 km.

#### Pregnancy Data

Women were asked the date of their last menstrual period and the date of the delivery of their baby. We calculated when in pregnancy women left and returned using pregnancy and temporary childbirth migration dates.

#### Socio-demographic Data

All socio-demographic data was merged in from routine data collection through the HDSS. Age was categorized into 19–24 (ref), 25–29, 30–34, 35–39 and 40 +; household income into less than 10,00 INR, 10,000–20,000 INR, 20,000–40,000 INR and over 40,000 INR; education into primary or less (ref), secondary, higher secondary, graduate and postgraduate, and occupation into housewife vs other. Parity was binary of first birth compared to all others. Sex of the baby (boy or girl) was included; women with twins were dropped from the regression analyses that included sex of the baby. We also included a variable for religion, which was made into a binary of Hindu compared to all others (Muslim, Buddhist, other) due to small sample size in all non-Hindu categories.

### Data Analysis

First, we explore patterns of temporary childbirth migration by departure and return date. Next, we describe the sociodemographic characteristics of our study population and compared migrators versus non-migrators using t-tests for continuous variables and chi-square tests or Fisher’s exact tests for categorical variables. We then estimate a series of logistic regression models to understand the association between sociodemographic characteristics and migration. We conduct sensitivity analyses to explore different patterns of migration by duration and time of departure. Data were analyzed using STATA version 17.0 (StataCorp, 2017).

## Results

### Migration Patterns

**Fig. 1:**
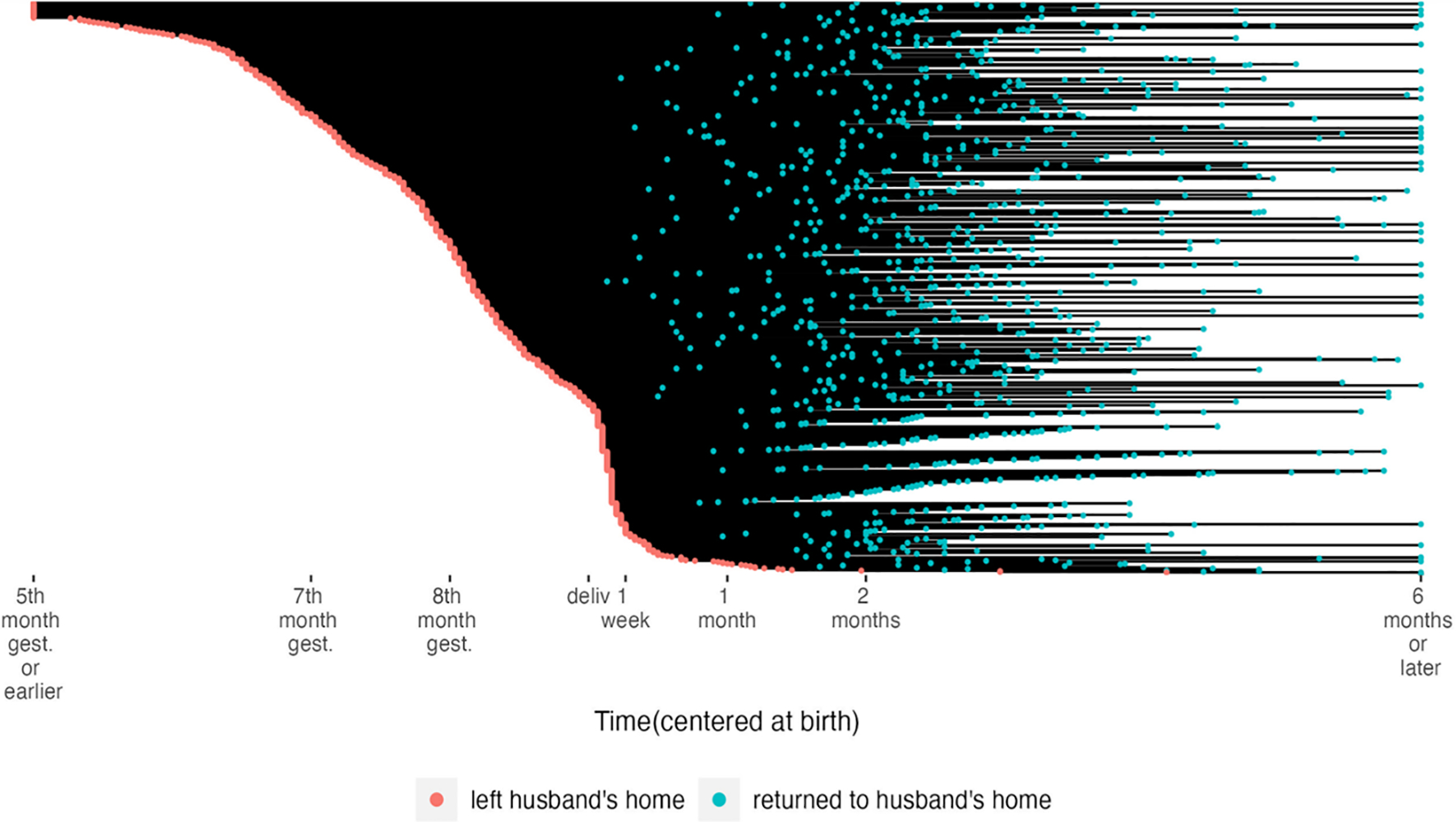
Plot of migration departure and return for all women

We visualized migration patterns in two ways. The first shows the duration of migration, graphed by when in the perinatal period a woman left for her natal home (Fig. 1). Some women left as early as the 5th month of pregnancy (few before that), but the frequency of departure for the natal home increased around 6 months, and then again around the 8th month. There was another bump in the number of departures right around the time of delivery, within the week following. Few women left later than 2 weeks after delivery. No clear trend emerged between when women left and when they returned, however, most women stayed at their natal home through birth. Around 2 months postdelivery there seems to be a thinning out of women in their natal home, although some do stay until after 6 months postpartum.

**Fig. 2:**
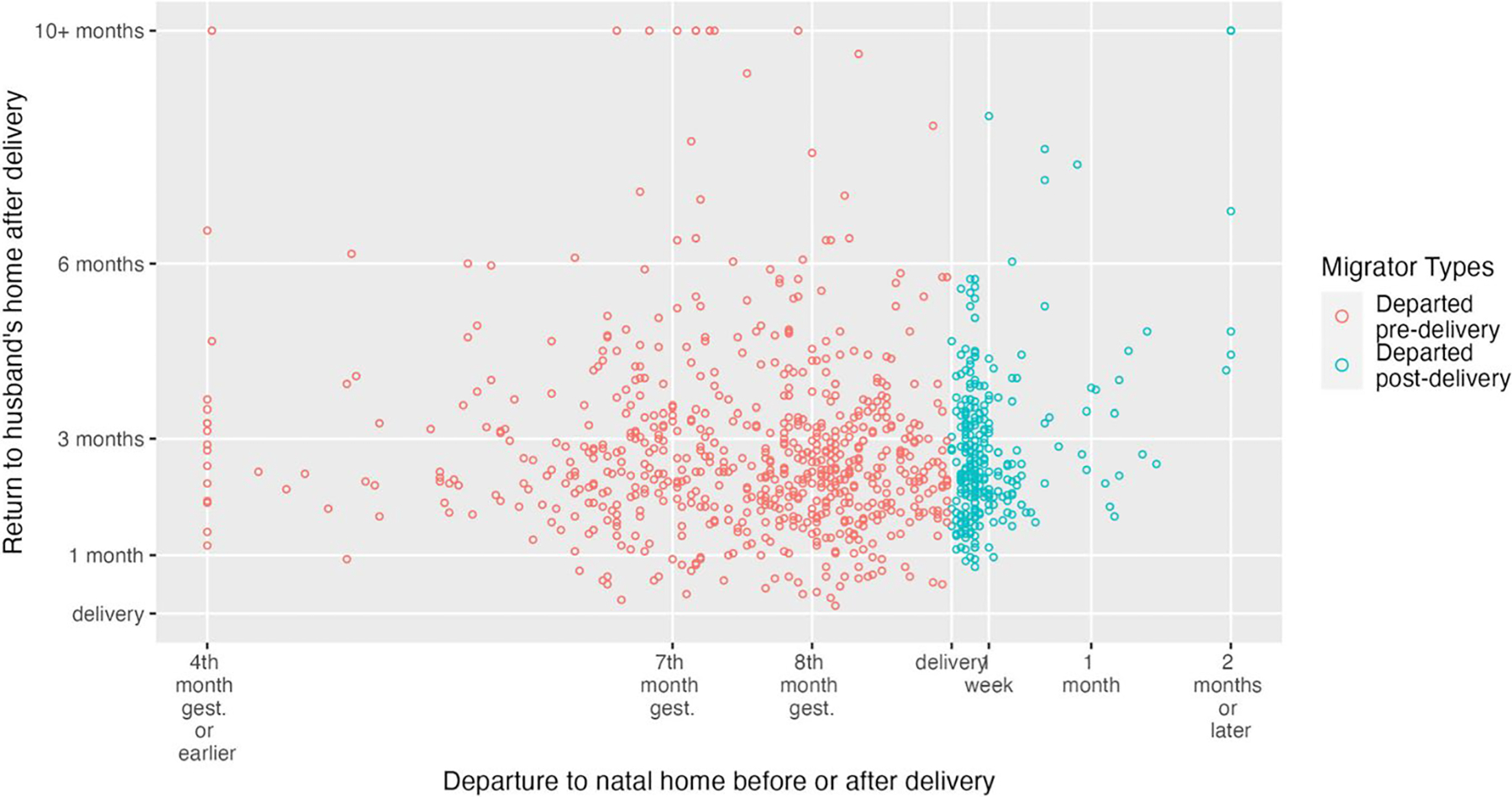
Plot of departure from husband’s home by return to husband’s home

We also visualized patterns by graphing departure date (x-axis) by return date (y-axis), to see if this allowed us to better identify patterns (Fig. 2). We can more clearly see in this Figure that most women returned home around the 2nd or 3rd month after delivery, regardless of when they departed. There appear to be two clusters of women: (1) those who left around the 8th month of pregnancy and who returned around 2nd or 3rd month months postpartum and (2) women who left right around/just after delivery and who returned around that same time (2nd or 3rd month postpartum).

### Temporary Childbirth Migration-Timing and Length

Out of the 1242 women surveyed, 991 (80%) migrated to their natal home for more than 1 month (Table 1). Most (81%, N = 1006) women returned to their natal home for more than 2 weeks, our original cut-off for temporary childbirth migration in our survey. Given the above, we defined temporary childbirth migration as migrating for more than 4 weeks (1 month) for the remainder of our analysis. We rationalized that this cutpoint would capture most women who migrated, as it was more conservative in excluding women who simply were visiting their parents for a prolonged trip of 2 or 3 weeks. Additionally, given the theorized impact on health care access/use, we hypothesized that a longer duration of migration would be needed for an interruption in care. With this cutoff in mind, we looked at duration by departure time, with a specific focus on those that migrated before delivery (in pregnancy, “pre-delivery migrators”) and those that migrated after delivery (post-partum, “post-delivery migrators”). About 40% of women migrated in the 8th or 9th month of pregnancy, with the next most common times being within 1 week after delivery (21.9%) and the 7th month of pregnancy (20.6%). Similar percentages migrated in the second trimester (7.1%) and 2–4 weeks after delivery (5.1%). Only 1.1% migrated in the first trimester and 1.7% more than 1 month after delivery. A few women (N = 6, 0.6%) migrated on the delivery date. Among all women, most stayed 2–4 months (48.9%), followed by 4–6 months (24.3%). Women who left earlier in pregnancy stayed longer. Very few women who left in pregnancy did not stay for delivery (N = 4, 0.4%).

### Distance to Natal Home and Impact on Timing and Duration

As can be seen in Table 2, about a third of women (N = 351, 35%) migrated over 100 km, with 8.5% migrating 50–100 km, 31.1% 20–50 km, 19% 5–20 km and only about 7% under 5km. Women who had to travel farther were more likely to migrate longer, when TCM was defined as being at the natal home for at least at least 3 months (OR 1.05 for those migrating over 100 km), however, there was no difference using the 1 month definition. Women who had to migrate farther were also more likely to go during pregnancy vs. postpartum (OR 1.60 for those migrating over 100 km). The impact of distance on migration patterns seemed to really make an impact for those migrating very far (over 100 km).

### Temporary Childbirth Migration and its Association with Socio-demographic Characteristics

Women in our sample ranged in age from 19 to 42, with a mean age in the full sample of 26.5 (Table 4). Women in younger age groups were more likely to migrate than women in older age groups (p = 0.015, when compared categorically) (Table 3). The mean parity in our full sample was 1.64. Women who were pregnant with their first birth were more likely to migrate, with almost 50% of those migrating having their first birth compared to 38% of those not migrating (p = 0.001). Over half (N = 649, 53.42%) had a boy, 43.46% (N = 528) a girl, and 3.13% (N = 38) twins, with no significant difference in migration by sex of the baby. Few of the sample had less than primary education (10%), 24% had secondary, 35% higher secondary, 26% graduate and almost 5% post-graduate education. The mean monthly income of the family was 24,855 INR, which falls into the lower income category. Women belonging to wealthier families and more educated women were also more likely to migrate (p < 0.0001 for both variables). Most women were Hindu (87.5%). There were no differences in migration pattern by religion or occupation.

Different socio-demographic characteristics were associated with migrating at different times in the perinatal period (Table 4). Compared to the youngest (reference) group, being an older woman was associated with being less likely to migrate longer than 1 month (OR − 0.81 95% CI 0.67–0.98), but age was not associated for those in the pregnancy period, only those postpartum. Similarly, education was associated with increased odds of temporary childbirth migration (OR 1.70, 95% CI 1.45–1.99), but when subgroup analyses were run, we see that higher educational attainment was more strongly associated with temporary childbirth migration in the postpartum period, however, still associated in the pregnancy period. Religion (not being Hindu) was associated with increased likelihood of migration in the pregnancy period, but not in the postpartum period (OR 1.81, 95% CI 1.26–2.61).

As a sensitivity analysis, we also looked at the narrower definition of temporary childbirth migration, defined as migrating for 3 months or longer. This measure aligns with the migration literatures definition of temporary migration (10). Just about half (N = 617, 49.7%) of women migrated for 3 months or longer. When we looked at socio-demographic variables associated with this more conservative definition of temporary childbirth migration, we found that the overall relationship with education held (OR 1.34 95% CI 1.18–1.52), however, now parity was inversely associated with temporary childbirth migration (higher parity associated with lower odds of temporary childbirth migration, OR 0.67, 95% CI 0.51–0.88) and age was no longer significantly associated. Additionally, non-Hindus were more likely to migrate with this longer definition (OR 1.56, 95% CI 1.10–2.21).

Across all models, income, sex of the baby and occupation were not significantly associated with migration patterns.

## Discussion

Our study provides insights into the migration patterns of a general population of rural Indian women residing in western rural Maharashtra in relation to childbirth and their associated demographic, social and economic factors. Temporary childbirth migration is clearly common, especially in this part of India. Women living in villages in this part of Maharashtra, Vadu, a few hours from Pune, often return to their natal home in pregnancy, for delivery, and into the postpartum period. When defined by 1 month or longer, 80% of women migrate, even with a more conservative measure of 3 months or longer, we find that half of women migrate in pregnancy. This is higher than rates found in different states of India (Bihar and Madhya Pradesh), which were closer to 30%, however, aligned with anecdotal evidence from other scholars working in India who estimated that around 70% of women migrated (Uttar Pradesh) (15). Clearly, this phenomenon is common and differs across India. The studies focused on it thus far have been in central India (Bihar and Madhya Pradesh) and among women who were migrants to urban areas in Mumbai (with the anecdotal evidence from Uttar Pradesh, which is northern India). Additional research in more states and among different castes, religions, urban/rural zone, etc., would be helpful in understanding more about the geographic heterogeneity of this practice.

A surprising finding of this analysis is that a substantial proportion of women did not migrate until after delivery, and they did so in the first week or so after their baby was born. Previously we had been conceptualizing this phenomenon as being something that occurred prior to delivery. With this new insight, we must consider how to appropriately gather data about and provide care for women and their infants in the postpartum period if they are at another location. The WHO postpartum care recommendations highlight that interruptions in the continuity of care in the post-partum period are just as important as in the prenatal period (World Health Organization, 2022). Furthermore, depending on distance between husband’s home/hospital and natal home, and means of transport (car, public transport, foot, etc.) this could potentially be putting newborns and recently delivered women at risk. Just under half of our sample migrated over 50 km (and about 35% over 100 km). This is quite a long distance for a pregnant woman or woman with a newborn to travel, especially given rural terrain in this area. We do see that women who have farther to go are more likely to leave in pregnancy and stay longer, suggesting that women may be taking into account things like health risks of travel when determining their migration timing and duration.

Temporary childbirth migration, as defined by either a 1 month or 3-month cut-off, impacts a large number of women and their infants in the focal area of research in this study, near Pune, Maharashtra, India. Given the length of most temporary childbirth migration, it easily fits within prior definitions of migration, if measured by length of time away. Additionally, since women may have multiple births in their lives, it also could be seen as a form of circular migration, although later parity women are less likely to migrate. However, women who migrate for childbirth are clearly different from other types of short-term migrants. For example, past research in India has suggested that short term migrants are generally less well off, however, we see higher education being predictive of this type of migration (Bala, 2017). However, choice of where to deliver has been found to be associated with a range of socio-demographic factors including education, parity, and migration status (Jeyashree et al., 2018; Kusuma et al., 2013).

By exploring migration by time in the peripartum period, our finding that no socio-demographics analyzed in this study were associated with migration in pregnancy suggest that this might be a cross-cutting cultural practice in this area. However, there are differences in who starts their migration in the postpartum period, with these women being more likely to be more highly educated. This suggests that perhaps more empowered or more well-off women (if education is a proxy for empowerment or wealth) might be able to advocate for leaving postpartum, to get that added support and rest in their natal homes. Prior theory has suggested that resources such as education can empower women to have more agency and then be able to act upon their desires (Kabeer, 1999).

While parity is not associated with the temporary childbirth migration using the 1-month definition, when we expand the definition of temporary childbirth migration to be 3 months or longer, we see that lower parity women are more likely to migrate, reflective of the common perception that this is a phenomenon mainly for women’s first birth. Our findings suggest therefore that women of all parities migrate, however primiparous women stay longer at their natal home. This is likely because the first birth has a special cultural/social significance and women may be “allowed” longer time to rest at the natal home, women might be perceived to need (or they themselves might want) more help, advice and support from their natal families, and/or there are fewer other responsibilities at the husbands home, such as prior children who also need care, pulling her back to her husband’s home sooner.

One explanation for the timing of migration we observed lies in socio-cultural traditions that occur at specific times in pregnancy and postpartum. Specifically, there is a tradition of having a baby shower in 7th month of pregnancy, and thus, one driver of the timing of leaving could be that women might wait to do the baby shower at their husband’s home and then return to their natal home, aligning with the increase in migration around 8 months of pregnancy. On the other end, a name ceremony generally occurs, again at the husband’s home, when the baby is 5 weeks old. This could be a driver behind the timing of return migration back to the husband’s home. There are some other socio-cultural factors that clearly play a role here as well, since religion (not being Hindu) was associated with migration with the 3-month definition, and earlier migration (in pregnancy). Given the small proportion of our sample that was not Hindu, we were not able to disentangle more about the role of religion in this practice; other studies with more diverse samples could provide insight. The role of tradition and cultural norms in the decision about when and if to migrate align with other evidence on the role of social and gender norms on a range of health behaviors in India (and globally) (Agha et al., 2021; Babbar et al., 2023; Kantor, 2002).

While much research is going on about migration, including more of a focus on women, migration due to non-labor or non-marriage related factors is absent. Clearly, temporary childbirth migration represents a common form of migration in a sensitive period for maternal and child health. We might ask why this common practice impacting millions of women and newborns a year, and occurring a few times in a woman’s lifetime potentially, is so under researched. Data is not collected on this phenomenon, perhaps due to lack of awareness of this practice or perhaps due to undervaluing of women and their experiences. A first step to remedying this inequity is collecting data on this practice—we need to know more about migration practices and the impact on health, and design solutions to ensure that women and newborns are getting the care that they need. To give a sense of the potential implications on current data collection and misclassification in this one state of India, we conducted a back of the envelope calculation. There are 131.6 million people in Maharashtra currently. The birth rate in 2020 was 15/1000, so this equates to 1,974,000 births a year. If 70% of these births belong to women who have migrated before delivery (as was found in our study), and therefore are delivering outside of where they usually live, this would mean that 1,381,800 births in this one state are misclassified each year. Of course, we do not know if people deliver in a different facility just because they are living somewhere else, but we do know about distance to the natal home. Most (84%) of women said that their natal home was more than 50km away, so if we assume that means they delivered at a different facility, then we would estimate that 1,160,712 births are misclassified in this one state each year. With increased migration generally, and especially for marriage (Mazumdar et al., 2013), and ease of transportation, more women may be away from their natal homes in the perinatal period, and thus, temporary childbirth migration might increase in the coming years.

Strengths of this analysis include providing evidence of the magnitude and duration of the understudied and common phenomenon of temporary childbirth migration using data from a large population of women. However, there are several limitations. Data was collected only from rural women, and collected retrospectively, which might lead to recall bias about exact dates of leaving and returning. Additionally, data was only collected from women who gave birth, thus we are missing information about women with adverse pregnancy outcomes. Another limitation is around the interpretation of these results in light of potential endogeneity. Since families choose who their daughter/son’s marry and might be considering both factors such as distance to the potential partner’s parents household and the other family’s willingness to let the woman migrate while pregnant/for delivery, ultimately distance and migration might be correlated due to marriage selection.

## Conclusion

In conclusion, our study sheds light on the significant phenomenon of temporary childbirth migration among Indian women residing in western rural Maharashtra. It highlights the prevalence of this practice, with a substantial proportion of women returning to their natal homes for childbirth and the postpartum period. This migration pattern is influenced by various demographic, social, and economic factors, and it varies across different regions of India. As many women migrate after childbirth, particularly within the first week following delivery it emphasizes the need to consider postpartum care and potential risks associated with such migrations, depending on the distance, means of transport and availability of healthcare facility.

Temporary childbirth migration affects a significant number of women and infants in India. However, it is concerning that this common practice, impacting millions of women and newborns each year, has been under-researched. The lack of data collection on this phenomenon may stem from a lack of awareness or undervaluation of women’s experiences. Addressing this research gap is crucial to understanding the impact of migration on maternal and child health and designing appropriate solutions. Including temporary childbirth migration, defined as a woman returning to her natal (parent’s) home for at least 1 month at any point in the pregnancy, childbirth or postpartum period, as a standard form of migration and understanding how it fits into traditional demographic migration theory is needed.

## Figures and Tables

**Table 1:** Temporary childbirth migration timing and length

	All migrators		Categories of migrators by departure and return timing																	
			Non-delivery migrators		Pre-delivery migrators, by gestational age at departure								Post-delivery migrators, by time passed since delivery							
					First trimester		Second trimester		7 months		8–9 months		On delivery date		Within 1 week of delivery		2–4 weeks after delivery		1 + month after delivery	
	n	*%*	n	*%*	n	*%*	n	*%*	n	*%*	n	*%*	n	*%*	n	*%*	n	*%*	n	*%*
All migrators*	991	100	4	0.4	11	1.1	69	7.1	201	20.6	403	41.3	6	0.6	214	21.9	50	5.1	17	1.7
By duration of migration																				
1–2 months	147	14.8	0	0	0	0	0	0	0	0	41	10.2	2	33.3	69	32.2	26	52	6	35.3
2–4 months	485	48.9	2	50	0	0	3	4.3	53	26.4	262	65	3	50	127	59.3	19	38	10	58.8
4–6 months	241	24.3	2	50	0	0	29	42	111	55.2	77	19.1	1	16.7	17	7.9	2	4	1	5.9
6–8 months	71	7.2	0	0	0	0	24	34.8	25	12.4	18	4.5	0	0	0	0	3	6	0	0
Greater than 8 months	47	4.7	0	0	11	100	13	18.8	12	6	5	1.2	0	0	1	0.5	0	0	0	0

**Table 2:** Migration description and association between migration distance and timing and duration of migration (among those who migrated, N = 1004)

	Proportion migrated each distance	Logistic regression models		
		TCM defined as 1 month	Migrating in pregnancy (compared to only migrating postpartum)	TCM defined as 3 months
	N (%)	OR (95% CI)	OR (95% CI)	OR (95% CI)
Distance of migration				
<5 km	66 (6.6%)	(Comparison)	(Comparison)	(Comparison)
5–20 km	190 (18.9%)	−0.042 (−2.32 to 2.24)	−0.10 (−0.67 to 0.47)	0.26 (−0.30 to 0.82)
20–50 km	312 (31.1%)	−0.40 (−2.51 to 1.71)	0.17 (−0.37 to 0.71)	−0.11 (−0.64 to 0.42)
50–100 km	85 (8.5%)	0.26 (−2.53 to 3.05)	0.57 (−0.11 to 1.25)	0.55 (−0.11 to 1.20)
Over 100 km	351 (35.0%)	0.58 (−1.70 to 2.86)	1.60*** (1.03 to 2.18)	1.05*** (0.51 to 1.59)
Constant		4.17*** (2.20 to 6.15)	0.31 (−0.18 to 0.79)	0.061 (−0.42 to 0.54)
Observations		1004	992	1004

*** p < .001

** p < .01

* p < .05

**Table 3:** Temporary childbirth migration by demographics

	Full sample (n = 1242)		Not migrated (n = 251)		Migrated (n = 991)		p-value
Age [mean, SD]	26.5	4.0	26.9	4.4	26.4	3.8	.081
Age group [n, %]							.015
19–24 years	418	33.7	83	33.1	335	33.8	
25–29 years	555	44.7	99	39.4	456	46	
30–34 years	221	17.8	52	20.7	169	17.1	
35–39 years	44	3.5	15	6	29	2.9	
40 + years	4	0.3	2	0.8	2	0.2	
Marital status [n, %]							
Married	1239	99.8	251	100	988	99.7	n/a
Unmarried	3	0.2			3	0.3	
Parity [mean, SD]*	1.64	0.71	1.85	0.86	1.59	0.65	n/a
Parity [n, %]							
Index was first birth	585	47.1	95	37.8	490	49.4	.001
2 + births	657	52.9	156	62.2	501	50.6	
Sex of the baby							
Male	649	53.42	128	52.03	512	53.77	.802
Female	528	43.46	109	44.31	419	43.24	
Twins	38	3.13	9	3.66	29	2.99	
Monthly income (INR) [mean, SD]	24,855	17,802	23,390	11,206	25,226	19,101	n/a
Monthly income (INR) [n, %]							< .0001
Less than 10,000	14	1.1	9	3.6	5	0.5	
10,000–20,000	353	28.4	82	32.7	271	27.3	
20,000–40,000	730	58.8	135	53.8	595	60	
40,000 or greater	145	11.7	25	10	120	12.1	
Education level [n, %]							< .0001
Primary or less	124	10	55	21.9	69	7	
Secondary	295	23.8	74	29.5	221	22.3	
Higher secondary	438	35.3	75	29.9	363	36.6	
Graduate	326	26.2	41	16.3	285	28.8	
Post-graduate	59	4.8	6	2.4	53	5.3	
Religion							.177
Hindu	1066	86.81	208	84.21	858	87.46	
Other (Muslim, Buddhist, other)	162	13.19	39	15.79	123	12.54	
Occupation [n, %]							
Housewife	1161	93.5	238	94.8	923	93.1	.571
Other	81	6.5	13	5.2	68	6.9	

*Notes* Independence for continuous variables assessed using two-sample t-test (assuming unequal variances for age). Independence for categorical variables assessed using Chi-Square

**Table 4:** Association between socio-demographic variables and temporary child migration (TCM) at different times in the perinatal period and using different definition (Odds Ratio, 95% confidence Intervals)

	Any TCM 4 weeks or longer	Any TCM 3 months or longer	Any TCM 4 weeks or longer, started in pregnancy	Any TCM 4 weeks or longer, started postpartum
Age (Categorical)^$^	0.81*	0.99	0.92	0.79*
	(0.67–0.98)	(0.84–1.16)	(0.78–1.07)	(0.65–0.96)
Parity	0.93	0.67**	0.82	0.92
	(0.67–1.31)	(0.51–0.88)	(0.63–1.07)	(0.65–1.30)
Sex of the baby	0.99	0.87	0.95	1.06
	(0.74–1.33)	(0.69–1.10)	(0.75–1.20)	(0.79–1.44)
Income (Categorical)	1.10	1.06	0.91	1.13
	(0.86–1.41)	(0.87–1.28)	(0.75–1.11)	(0.88–1.45)
Education (Categorical)	1.70***	1.34***	1.13*	1.74***
	(1.45–1.99)	(1.18–1.52)	(1.00–1.28)	(1.48–2.05)
Occupation (housewife vs other)	1.39	0.65	0.69	1.49
	(0.72–2.68)	(0.40–1.05)	(0.43–1.10)	(0.75–2.96)
Religion (categorical)	0.89	1.56*	1.81**	0.84
	(0.59–1.34)	(1.10–2.21)	(1.26–2.61)	(0.56–1.28)
Constant	0.85	1.24	2.73*	0.71
	(0.25–2.92)	(0.48–3.18)	(1.07–6.98)	(0.20–2.53)
Observations^#^	1,177	1,177	1,177	1,177

$ Age was categorized into 19–24 (ref), 25–29, 30–34, 35–39 and 40 +; household income into less than 10,00 INR, 10,000–20,000 INR, 20,000–40,000 INR and over 40,000 INR; education into primary or less (ref), secondary, higher secondary, graduate and postgraduate, and occupation into household vs other. Parity was categorial of first birth compared to all others. Sex of the baby is boy (ref) compared to girl. Religion was binary of all others compared to Hindu

# N reduced because all twins were dropped from the analysis

*** p < .001

** p < .01

* p < .05

## Data Availability

Data will be made publicly available within 1 year of the completion of data collection both through the Vadu HDSS and UCSF.
